# Predictive ability of viscoelastic testing using ClotPro® for short-term outcome in patients with severe Covid-19 ARDS with or without ECMO therapy: a retrospective study

**DOI:** 10.1186/s12959-022-00403-0

**Published:** 2022-08-29

**Authors:** Lars Heubner, Marvin Greiner, Oliver Vicent, Jan Beyer-Westendorf, Oliver Tiebel, Ute Scholz, Andreas Güldner, Martin Mirus, Dietmar Fries, Thea Koch, Peter Markus Spieth

**Affiliations:** 1 Department of Anesthesiology and Intensive Care Medicine, University Hospital “Carl Gustav Carus”, Technische Universität Dresden, Dresden, Germany; 2 Division of Hematology and Hemostasis, Department of Medicine I, Thrombosis Research University Hospital “Carl Gustav Carus”, Technische Universität Dresden, Dresden, Germany; 3 Institute of Clinical Chemistry, University Hospital “Carl Gustav Carus”, Technische Universität Dresden, Dresden, Germany; 4 MVZ Labor Dr. Reising-Ackermann Und Kollegen, Center of Hemostasis, Leipzig, Germany; 5 Department for General and Surgical Critical Care Medicine, Innsbruck Medical University, Innsbruck, Austria

**Keywords:** COVID-19, ARDS, Coagulopathy, Fibrinolysis, Hemostatsis, Viscoelastic testing

## Abstract

**Background:**

SARS-CoV-2 infections are suspected to trigger the coagulation system through various pathways leading to a high incidence of thromboembolic complications, hypercoagulation and impaired fibrinolytic capacity were previously identified as potentially mechanisms. A reliable diagnostic tool for detecting both is still under discussion. This retrospective study is aimed to examine the prognostic relevance of early viscoelastic testing compared to conventional laboratory tests in COVID-19 patients with acute respiratory distress syndrome (ARDS).

**Methods:**

All mechanically ventilated patients with COVID-19 related ARDS treated in our intensive care unit (ICU) between January and March 2021 were included in this study. Viscoelastic testing (VET) was performed using the ClotPro® system after admission to our ICU. Prevalence of thromboembolic events was observed by standardized screening for venous and pulmonary thromboembolism using complete compression ultrasound and thoracic computed tomography pulmonary angiography at ICU admission, respectively. We examined associations between the severity of ARDS at admission to our ICU, in-hospital mortality and the incidence of thromboembolic events comparing conventional laboratory analysis and VET. ECMO related coagulopathy was investigated in a subgroup analysis. The data were analyzed using the Mann–Whitney U test.

**Results:**

Of 55 patients enrolled in this study, 22 patients required treatment with ECMO. Thromboembolic complications occurred in 51% of all patients. Overall hospital mortality was 55%. In patients with thromboembolic complications, signs of reduced fibrinolytic capacity could be detected in the TPA assay with prolonged lysis time, median 460 s (IQR 350–560) vs 359 s (IQR 287–521, *p* = 0.073). Patients with moderate to severe ARDS at admission to our ICU showed increased maximum clot firmness as a sign of hypercoagulation in the EX-test (70 vs 67 mm, *p* < 0.05), FIB-test (35 vs 24 mm, *p* < 0.05) and TPA-test (52 vs 36 mm, *p* < 0.05) as well as higher values of inflammatory markers (CRP, PCT and IL6). ECMO patients suffered more frequently from bleeding complications (32% vs 15%).

**Conclusion:**

Although, the predictive value for thromboembolic complications or mortality seems limited, point-of-care viscoelastic coagulation testing might be useful in detecting hypercoagulable states and impaired fibrinolysis in critically ill COVID-19 ARDS patients and could be helpful in identifying patients with a potentially very severe course of the disease.

## Background

Since the outbreak in late 2019,COVID-19 has been spreading worldwide and was declared a global pandemic on March 11, 2020 by the World Health Organization (WHO) [1]. Already during the first wave in spring 2020 limited evidence suggested that the severity of COVID-19 associated acute respiratory distress syndrome (cARDS) cannot solely be explained due to inflammation alone. In fact, clinical and laboratory findings confirmed the role of autoimmune processes resulting in micro- and macrovascular thromboses in the pathogenesis of the disease [2–6] early on. The interplay between infection, inflammation, immune response on one side and stimulation of the coagulation system on the other has been recognized for a long time [7–9]. In COVID-19 endothelial damage of both the respiratory and vascular endothelium appears to lead to a pronounced coagulation activation in the lung. As a result, lung imaging of severely ill COVID-19 patients demonstrates local thromboses of small and large pulmonary arteries that are distinctively different from pulmonary emboli. Instead of a random distribution pattern these COVID-19 lung thrombi are often restricted to lung areas most affected by the viral infection [10]. This indicates that, apart from systemic hypercoagulability [11], local hypercoagulability in the lung vasculature might have a major impact on functional outcome and mortality in cARDS [12–14]. The mechanisms of this hypercoagulable condition are various. SARS-CoV-2 is directly bound by angiotensin-converting enzyme 2 (ACE-2) receptors, resulting in a strong activation of tissue factor (TF), exposure of collagen and release of von Willebrand factor (vWF) [11]. TF triggers the release of endotoxins and tumor necrosis factor α and activates the extrinsic coagulation pathway by interaction with factor VII. The exposed collagen, combined with antithrombin-III (ATIII), activates the intrinsic coagulation pathway. TF, collagen and vWF facilitate platelet activation and recruitment, followed by aggregation and clot formation. The intrinsic and extrinsic coagulation pathways cause fibrin formation, leading to a stable platelet–fibrin-clot. SARS-CoV-2 binding ACE-2 receptors leads to its downregulation [15] resulting in an accumulation of angiotensin-II and a severe imbalance in the renin–angiotensin–aldosterone system (RAAS), which can cause a cytokine storm [16]. In addition, it cannot be ruled out that COVID-19 can directly activate the thrombin generation by thrombin like proteases. Such a mechanism has been reported for H7N6 Avian Influenza Virus, which resulted in a hemagglutinin activation and increased virulence leading to an increase of thrombin generation and activation [17]. Although SARS-CoV-2 does not belong to the class of influenza viruses it is a striking observation that many of the severely ill COVID-19 patients demonstrate markers of thrombin overload as well [18, 19]. Further studies provide evidence that other complex pathophysiological mechanisms may contribute to the hyperinflammatory-hypercoagulable state in COVID-19. Coagulation factor XII (the “contact” factor), activated by pro-inflammatory mediators, plays an important role in the formation process of microthrombi linking coagulation processes to the bradykinin system. This most likely immune-mediated hypercoagulation differs from other sepsis-related coagulopathies [20, 21]. Furthermore, another pathway via endothelial activation seems to exist. Endothelial cells with their high levels of ACE-2 are major participants and regulators of coagulation [22]. Their dysfunction leads to the hypercoagulable state often seen in severe COVID19 cases.

Additionally, recent studies found high plasma levels of plasminogen activator inhibitor 1 (PAI-1), released from infected, activated endothelial cells and platelets in septic [23] and COVID-19 patients [18] associated with worse outcome [18]. PAI-1, emitted by monocytes, is a strong inhibitor of fibrinolysis [24]. Ranucci et al. showed that COVID-19 patients with worse outcome had up to sixfold higher PAI-1 levels compared to survivors [18]. Visceral fat has been reported to be the main physiological storage for PAI-1 [25] and higher PAI-1 values have been shown in obese patients. As a result of high plasma levels of PAI-1, fibrinolysis mediated by tissue plasminogen activator (tPA) and urokinase plasminogen-activator (uPA) may be severely reduced [26] and could lead to impaired fibrinolysis, which is frequently seen in COVID-19 patients [27–30] as well as in septic patients [31]. This could also explain why many obese patients develop a severe course of COVID-19. Early recognition of hypofibrinolysis is important but difficult to achieve with clinically established standard laboratory analyses. D-dimers, fibrinogen and C-reactive protein (CRP) are often used as surrogate parameters for increased risk of fibrinolytic shutdown [32–35] but they lack specificity.

**Fig. 1:**
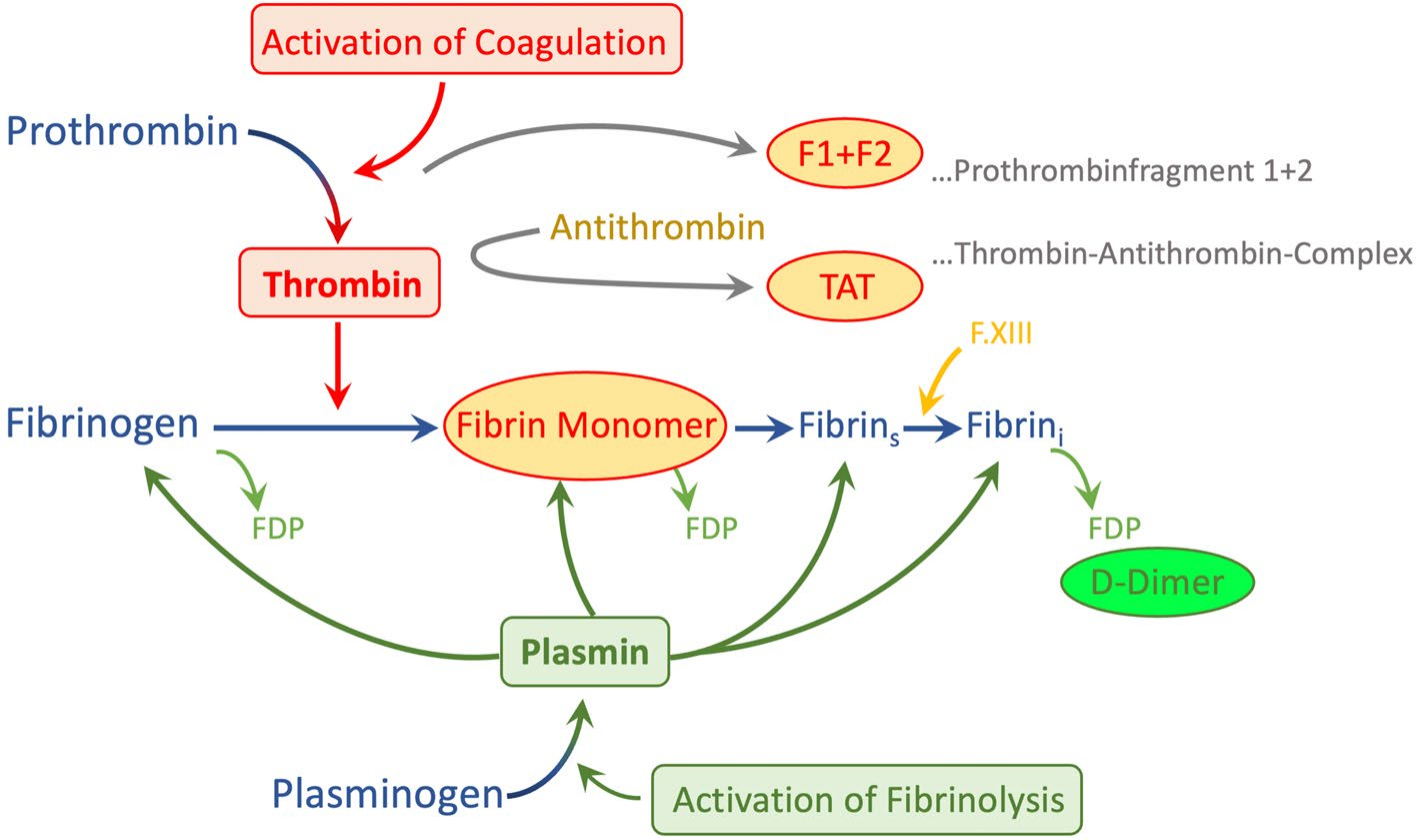
Diagnostic Markers for Coagulation and Fibrinolysis (Permission Dr. Tiebel)

There are some specialized coagulation tests to quantify increased coagulability and thrombin stimulation, such as prothrombin fragment 1 + 2 (PF 1 + 2) or decreased fibrinolysis such as PAI-1, tPA, Thrombin-Antithrombin-Complex (TAT) or plasmin-antiplasmin complex (PAP) [18]. Frequent analysis of PF 1 + 2 levels, which are directly proportional to thrombin generation in real time, could help detecting increased clotting activation at an early stage (Figure 1). However, these analyses are currently not available for standard patient care in routine laboratories.

Therefore, viscoelastic testing (VET) as Point-of-care (POC) test for different but interrelated coagulation pathways under *in-vitro* conditions could be beneficial in clinical practice. Both extrinsic and intrinsic coagulation pathways as well as fibrinolysis can be evaluated in detail as follows: Clot formation and clot firmness as markers of hypercoagulation are assessed by maximum clot firmness (MCF) in ClotPro®. Clot lysis as a marker of impaired fibrinolysis is assessed by maximum lysis (ML) and lysis time (LT) in ClotPro® [28, 29, 36, 37]. A recently developed test with the addition of tPA to initiate fibrinolysis seems to be a promising technique to detect impaired fibrinolysis [38–40].

Recent studies showed a strong correlation between thromboembolic events and abnormal results in VET, in particular when combined with D-dimer analysis [29, 36, 41]. It remains unclear whether this could be helpful to predict or even prevent arterial or venous thromboembolic complications (ATE/VTE) by adjustment of anticoagulation therapy.

The aim of this study was to investigate the role of a POC VET (ClotPro®) compared to conventional laboratory analyses for evaluation of the coagulation system in mechanically ventilated COVID-19 patients with severe ARDS and high risk for ATE/VTE. In a first step, we analyzed the correlation between ARDS severity and the results of VET and conventional laboratory assays as well as patient individual characteristics at admission to our intensive care unit (ICU). In a second step, the prediction of different endpoints was analyzed using results of VET and conventional laboratory results at admission to our ICU. The first endpoint was the prevalence of any ATE/VTE during the entire ICU stay, the second endpoint was defined as in-hospital mortality and the third endpoint was the prediction of bleeding events. The third aim of this study was to investigate the correlation between conventional laboratory testing and VET for anticoagulation monitoring in severely ill patients. Therefore, we investigated the correlation between: 1) Prothrombin time (displayed as international normalized ratio) and clotting time in EX-test as assessments of the extrinsic coagulation pathway, 2) activated partial thromboplastin time and clotting time in IN-test as assessments of the intrinsic coagulation pathway and 3) plasma fibrinogen value and maximum clot firmness in FIB-test as measures for fibrinogen values. ECMO related coagulopathy was additionally investigated in a subgroup analysis comparing VTE/ATE as well as bleeding complications between patients with and without ECMO therapy.

## Methods

### Study design

We performed this retrospective single-center study in a tertiary ICU and ARDS/ECMO referral center at the University Hospital Dresden, Germany. The study was conducted in accordance with the Declaration of Helsinki and approved by the responsible Ethics Committee of the Technical University of Dresden, Germany (BO-EK-374072021). All patient data were recorded during the ICU stay using the standard electronic patient data management system (ICM, Dräger Medical). ATE/VTE were defined as a composite endpoint, consisting of DVT examination including the lower leg (including catheter associated events, CAT), pulmonary embolism (PE) and arterial events (myocardial infarction, stroke, systemic embolism or acute arterial thrombosis in peripheral or mesenterial arteries) during the ICU stay. Bleeding complications were defined according to the Bleeding Academic Research Consortium (BARC) type 2–5 definition [42]. BARC type 3–5 bleeding is classified as fatal bleeding or clinical, laboratory or imaging evidence of bleeding making specific interventions necessary. Additionally, BARC type 2 is defined as any clinical sign of apparent haemorrhage that does not fit the criteria for type 3 to 5. Given the long-term stay of patients and the frequent repetition of hemoglobin testing and blood transfusions, we calculated the BARC bleeding classification using hemoglobin values or number of red blood cell transfusions from 24 h after bleeding onset.

### Inclusion criteria

All patients admitted to the University Hospital Dresden with a SARS-CoV-2 infection confirmed by polymerase chain reaction presenting with severe respiratory failure and requiring invasive mechanical ventilation between January 2021 and March 2021 were included in this study.

### Laboratory analysis

Standard laboratory analyses included relative prothrombin time (PT in % of normal and INR), activated partial thromboplastin time (aPTT), fibrinogen, fibrin monomers and D-dimers on STA R Max3-Analyzers (STAGO Deutschland GmbH, Düsseldorf, Germany). PF 1 + 2 was analyzed applying LOCI-technology on an Atellica COAG 360 System (Siemens Healthcare GmbH, Erlangen, Germany).

Additional blood count analyses were performed using EDTA-tubes for hemoglobin concentration, white blood cell count and platelet count. A serum collecting tube was used for measurements of inflammatory parameters (CRP, Interleukin 2 and 6 (IL2, IL6), Procalcitonin (PCT) and organ function monitoring (creatinine, bilirubin, and albumin).

### Viscoelastic testing

At defined time points (the first Monday, Wednesday or Friday following admission to our ICU), blood samples for laboratory analyses, POC blood-gas-analysis and VET were drawn from each patient at the same time. VET was performed once for every patient at admission to our ICU. VET samples were processed for 40 min operating time under standardized conditions at 37 °C within a maximum of two hours after arterial blood sampling. ClotPro® (enicor, Munich, Germany) is a newly developed VET system that uses a cup and a pin to measure clot formation, with the cup rotating via an elastic element and the pin functioning as a stationary counterpart [43, 44]. The original technique was described by Hartert in 1951 [45]. Basically, the mechanical deceleration of the cup rotation is detected and translated into a viscoelastometric amplitude. ClotPro® is a bedside available POC device, mainly used in the ICU, operation room or emergency department. Technically, ClotPro® it is somewhat different from ROTEM® and TEG® because it has a fixed pin but a spinning cup and measuring the deceleration via an elastic element. The pipette tips contain the test specific reagent and are ready-to-use, improving the work flow and reducing process errors. Another feature is the flexibility of analyses: Out of a total spectrum of 8 available tests, up to six different tests can be performed simultaneously for a single patient (using all six channels of the device) or accordingly one test for up to six patients thus reducing the costs per patient. Probably the most important feature is that ClotPro® provides additional test options such as TPA-test, ECA-test, and RVV-test. The last two can be used for detection of direct thrombin inhibitors or factor Xa-antagonists, respectively [46]. The TPA assay contains recombinant tissue plasminogen activator and may be used to identify impaired fibrinolysis as a result of tranexamic acid [47] or due to intrinsic impaired fibrinolysis [40]. Of note, although ClotPro® results on EX-test and FIB-test seem to be comparable to ROTEM values, IN-test results differ in thresholds [48].

**Fig. 2:**
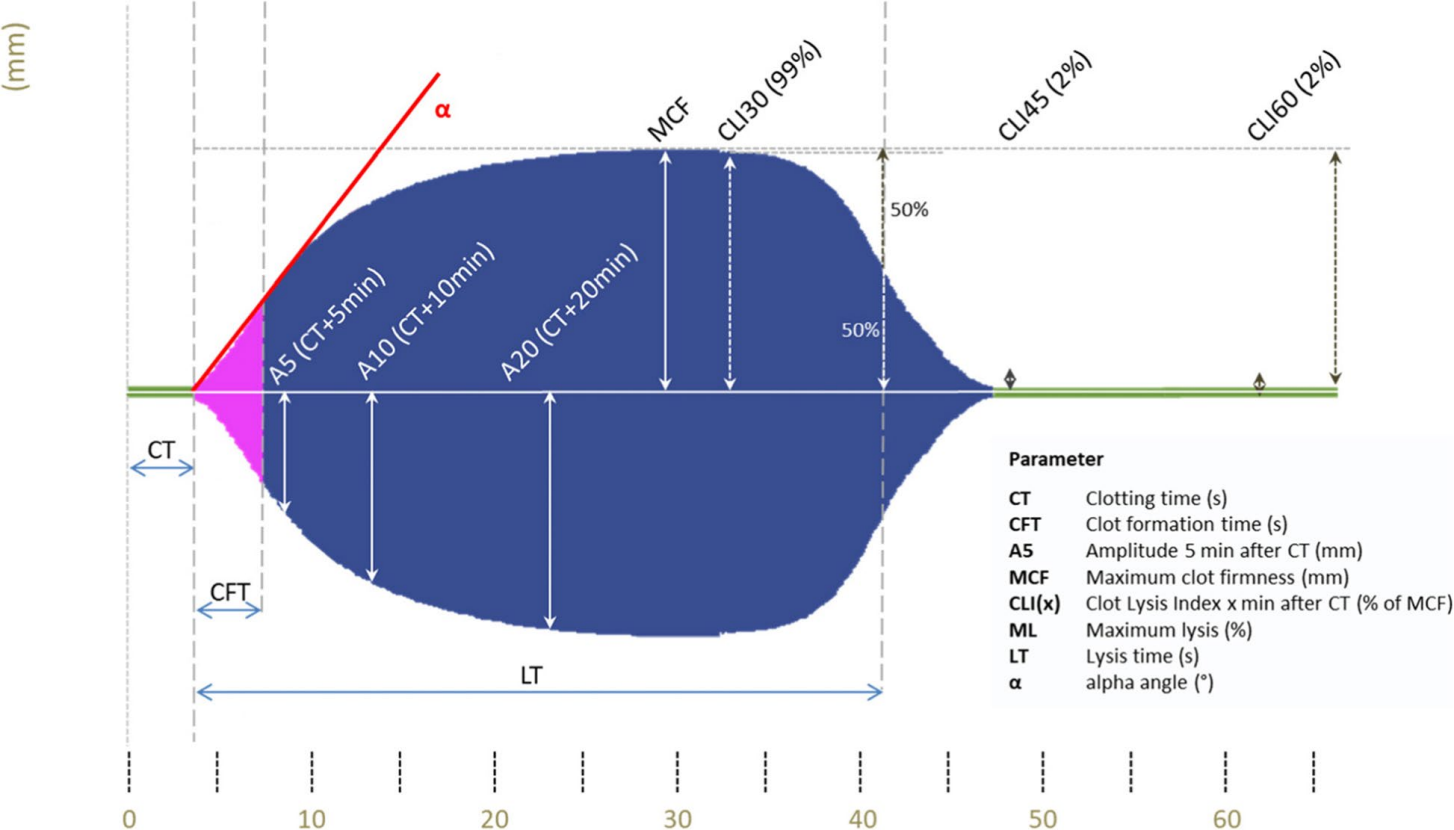
ClotPro® Parameters (Permission Haemonetics©)

Measurements were performed following the manufacturer’s guidelines using the test specific syringe for pipetting 340 µL of citrated patients’ blood per test and releasing it into the cups. Each assay’s specific pipette tip is loaded with the respective dried reagent. For the present study, we used EX- (assessment of extrinsic coagulation pathway), FIB- (examination of fibrinogen level and fibrin integration), IN- (assessment of intrinsic coagulation pathway) and TPA- (detection of hypofibrinolysis) test. EX- and FIB-tests are heparin-insensitive because hexadimethrine bromide is added, CaCI_2_ recalcifies the sample in both assays and recombinant tissue factor (rTF) starts the coagulation. FIB-test analyses clot formation after platelet inhibition by addition of cytochalasin D and a synthetic GP2b3a antagonist. IN-test also uses CaCI_2_ to recalcify the samples but ellagic acid for coagulation activation. In the TPA essay, recombinant tissue plasminogen activator (r-tPA; 650 ng/mL) is used to cleave plasminogen to plasmin as a potent activator of fibrinolysis. ClotPro® system provides various parameters (Figure 2) such as clotting time (CT; period of time from start to a 2 mm thickness of clot amplitude), clot formation time (CFT; time needed for clot amplitude from 2 to 20 mm), A5 (thickness of clot amplitude 5 min after CT), A10 (thickness of clot amplitude 10 min after CT), maximum clot firmness (MCF; overall maximum thickness of the clot amplitude), maximum lysis (ML; percentage of lysis in relation to MCF during the overall time of measurement) and lysis time (LT; period of time from CT until 50% lysis of clot formation). In case of complete fibrinolysis shutdown, when no lysis of 50% MCF was recorded in TPA-essay, LT was set as the maximum runtime of each viscoelastic test and 2400 s were used for statistical calculations.

### Anticoagulation therapy

All patients in our ICU were treated according to the same standard operating procedure (SOP) for anticoagulation therapy with consulting support by the department of internal medicine to evaluate the individual patient´s risk for thrombosis at the time of ICU admission. On ICU admission, all patients were screened for venous thromboembolism (VTE) using complete compression ultrasound (cCUS) SOPs. Preexisting pulmonary embolism (PE) was detected by thoracic computed tomography pulmonary angiography (CTPA). Further cCUS and CTPA were performed, if clinical signs of venous or arterial thrombosis or embolism occurred. If PE was newly diagnosed, cCUS screening was repeated. Patients without venous or arterial thromboembolism received standard weight-based sub-therapeutic unfractionated heparin (target aPTT of 40-50 s) or intermediate doses of low molecular weight heparin (100 aXa units/kg/d). This intermediate dose anticoagulation approach was chosen as standard anticoagulation for all critical ill COVID-19 patients, according to our institutional ICU-SOPs. All patients with confirmed ATE/VTE received therapeutic weight-based unfractionated heparin (target aPTT of 60-80 s) or low molecular weight heparin (200 aXa units/kg/d). Patients with contraindications for full therapeutic anticoagulation received a patient specific therapy, according to benefit-risk assessments which included thrombi burden, bleeding risk or current bleeding intensity. Anticoagulant treatment target for such patients was set according to aPTT of 50-60 s or LMWH dosages between 100–200 units/kg/d. This was defined as intermediate dose anticoagulation.

### ARDS and ECMO

ARDS severity was defined according to the Berlin definition with PaO_2_/F_I_O_2_ thresholds of < 300 mmHg for mild, < 200 mmHg for moderate and < 100 mmHg for severe ARDS. All patients with refractory severe hypoxemia fulfilling the EOLIA criteria [49] were evaluated for ECMO therapy. Individual treatment was decided in a multidisciplinary approach. ECMO was performed as femoro-jugular veno-venous bypass using percutaneous ultrasound guided insertion of drainage and return cannula.

### Statistical analyses

Statistical analyses were performed using the SPSS Statistics 27 software (IBM, Inc, Armonk, NY, U.S.) and R version 3.2.4. All categorical variables are described as absolute and relative frequencies; comparison between groups was done using Fisher's exact test. Continuous variables were presented as median and 1^st^; 3^rd^ quartile; group comparison was based on the Mann–Whitney U test. Correlations between viscoelastometric variables and conventional laboratory parameters were done by Spearman`s correlation. Significance level was set at 0.05.

## Results

### Clinical characteristics (baseline)

Between 01/2021 and 03/2021 55 patients were treated for severe respiratory failure secondary to COVID-19 infection in our ICU. Median age was 64 years (range 42–81) and 78% of the patients were male (*N* = 43, baseline characteristics are shown in Table 1). Due to the fact that the majority of our patients (*n* = 38, 62%) were pretreated in ICU of other hospitals, VET measurements were performed at median of 17 days (range 3–38) from onset of first symptoms. In all patients, ARDS was diagnosed with median PaO_2_/F_I_O_2_ of 146 mmHg (range 75–219, 114; 219) for non-ECMO patients at admission to our ICU. All patients developed severe ARDS during stay on our ICU with lowest median PaO_2_/F_I_O_2_ of 53 mmHg (range 38–98, 45; 60) for non-ECMO patients. In 40% (*n* = 22) veno-venous ECMO was necessary to maintain adequate gas exchange. The time between onset of symptoms and hospital admission was median 6 days (range 0–21), for ICU admission 10 days (range 0–38), for intubation 10 days (range 2–25) and for ECMO therapy 17 days (range 7–53). The majority of the patients had preexisting conditions (82%, *n* = 45) with a median Charlson comorbidity index of 3 points (range 0 – 10). Arterial hypertension (66%, *n* = 36) and diabetes (40%, *n* = 22) were frequent and obesity was common in this cohort (median BMI 28, range 19–70). All patients showed critical organ failure on the day of study enrollment with a median SOFA score of 11 (range 7–17). The majority of patients received an intermediate anticoagulation regime (47%, *n* = 26), 31% (*n* = 17) were therapeutically anticoagulated and 22% (*n* = 12) received prophylactic anticoagulation only. Unfractionated heparin was used for anticoagulation in 78% (*n* = 43) with median doses of 1500 IE/h (range 600–3000). In three patients (5%) systemic fibrinolytic therapy for PE was applied more than 72 h prior to admission to our ICU. Preexisting administration of platelet aggregation inhibitors were present in 10 patients (18%), whereas 1 patient (2%) received dual platelet inhibition.

**Table 1:** Baseline characteristics and survival

	**All patients**	**Survivors**	**Non-Survivors**	p
n	55	25 (45%)	30 (55%)	
Male	43 (78%)	17 (68%)	26 (87%)	0.090
Age [years]	65 (58; 69)	63 (57; 68)	68 (61; 71)	** < 0.05**
Body-Mass-Index [kg/m^2^]	28 (25; 36)	31 (25; 36)	28 (25; 31)	0.723
Time from first symptom to hospital admission [days]	6 (4; 10)	6 (4; 8)	7 (4; 10)	0.416
Time from first symptom to ICU admission [days]	10 (6; 14)	10 (6; 15)	9 (6; 14)	0.442
Time from first symptom to invasive ventilation [days]	10 (6; 15)	10 (6; 17)	11 (7; 14)	0.629
Time from first symptom to ECMO therapy [days]	17 (13; 23)	19 (11; 23)	16 (14; 21)	0.769
Time from first symptom to admission to our ICU [days]	12 (7; 17)	13 (8; 18)	12 (7; 16)	0.741
Nosocomial infection	8 (15%)	3 (12%)	5 (17%)	0.841
UFH dose [IE/h] ^*^	1500 (1100; 1900)	1600 (1100; 1900)	1450 (1050; 1750)	0.327
UFH dose [IE/kg/h] ^*^	17 (11; 20)	18 (14; 21)	15 (9; 20)	0.203
LMWH dose [mg/d] ^*^	120 (100; 180)	120 (80; 120)	130 (120; 180)	0.329
LMWH dose [mg/kg/d] ^*^	1.5 (1.1; 2)	1.2 (1.1; 1.2)	1.6 (1.5; 2.0)	0.429
Prophylactic ACT ^*^	12 (22%)	5 (20%)	7 (23%)	0.514
Intermediate ACT ^*^	26 (47%)	14 (56%)	12 (40%)	0.181
Therapeutic ACT ^*^	17 (30%)	6 (24%)	11 (37%)	0.237
VV-ECMO therapy	22 (40%)	12 (48%)	10 (33%)	0.269
Direct transfer to our ICU from other hospital	34 (62%)	17 (68%)	17 (57%)	0.389
Invasive mechanical ventilation before admission to our ICU	13 (24%)	13 (52%)	0	** < 0.05**
SOFA score [points] ^*^	11 (10; 13)	10 (9; 11)	12 (11; 14)	** < 0.05**
Horovitz – Index at admission to our ICU without patients under ECMO therapy [mmHg]	146 (114; 219)	204 (138; 220)	129 (111; 179)	0.118
ARDS level mild ^*^	11 (20%)	7 (28%)	4 (13%)	0.176
ARDS level moderate ^*^	19 (35%)	5 (20%)	14 (47%)	** < 0.05**
ARDS level severe ^*^	25 (45%)	13 (52%)	12 (40%)	0.373
Norepinephrine [mg/h] ^*^	0.5 (0.3; 1.1)	0.4 (0.15; 0.7)	0.8 (0.4; 1.4)	** < 0.05**
Norepinephrine [µg/kg/min] ^*^	0.09 (0.05; 0.24)	0.07 (0.02; 0.12)	0.14 (0.07; 0.31)	** < 0.05**
Charlson comorbidity index [points]	3 (2; 5)	3 (2; 5)	3 (2; 6)	0.148
Terminal renal insufficiency with need of Renal Replacement Therapy	3 (6%)	0	3 (10%)	0.147
Systemic thrombolysis before admission to our ICU	3 (6%)	1 (4%)	2 (7%)	0.569
Oral anticoagulation before hospital admission	5 (9%)	4 (17%)	1 (3%)	0.122
Single platelet inhibition before hospital admission	10 (18%)	5 (20%)	5 (17%)	0.510
Dual platelet inhibition before hospital admission	1 (2%)	0	1 (3%)	0.545
All Patients with complications related to coagulopathy*	31 (56%)	15 (60%)	16 (53%)	0.412
All patients with bleeding complications	10 (18%)	5 (20%)	5 (17%)	0.510
BARC-classification II	4 (7%)	2 (8%)	2 (7%)	0.622
BARC-classification III-V	8 (15%)	4 (16%)	4 (13%)	0.573
All ATE/VTE during hospital stay	27 (49%)	14 (56%)	13 (43%)	0.620
ATE/VTE diagnosed before admission to our ICU	22 (40%)	11 (44%)	11 (37%)	0.391
New ATE/VTE diagnosed during stay on our ICU	5 (9%)	3 (12%)	2 (7%)	0.412

^*^ at day of VET (equals admission to our ICU)

*ICU* Intensive Care Unit, *(VV-)ECMO*, (venovenous-)Extracorporeal Membrane-Oxygenation, *UFH* Unfractionated Heparin, *LMWH* Low-Molecular-Weight Heparin, *ACT* Anticoagulation Therapy, *cCUS* Complete Compression Ultrasound, *CTA* Computed Tomography Angiography, *SOFA* Sepsis-related Organ Failure Assessment, *ARDS* Acute Respiratory Distress Syndrome, *BARC* Bleeding Academic Research Consortium, *ATE/VTE* Arterial/Venous Thromboembolism, *VET* Viscoelastic Testing

### ARDS severity and global coagulation tests at admission to our ICU

At admission to our ICU 11 patients (20%) presented with mild ARDS, while 19 patients (35%) presented with moderate and 25 (45%) presented with severe ARDS. Patients with moderate to severe ARDS showed significantly higher SOFA-scores (12 vs 9 points), lower hemoglobin counts (5.7 vs 6.6 mg/dl), higher fibrinogen values (6.7 vs 4.1 g/l), higher CRP (186 vs 55 mg/l), PCT (1.35 vs 0.45 ng/ml) and IL-6 (186 vs 27 pg/ml) levels, respectively. Furthermore, aPTT was significantly prolonged in all patients with moderate or severe ARDS compared to mild ARDS (46 vs 30 s). Additionally, these patients presented with significantly increased values for clotting firmness as sign of hypercoagulability in MCF at EX-test (median 70 vs 67 mm), FIB-test (35 vs 24 mm) and TPA-test (52 vs 36 mm) compared to patients presenting mild ARDS at admission to our ICU. Notably, the time from the onset of first symptoms to measurement was numerically, but not significantly prolonged in patients presenting moderate to severe ARDS compared to patients with mild ARDS (18 vs 13 days). PF 1 + 2 and D-dimers did not differ between both groups (Tables 2 and 3).

**Table 2.**
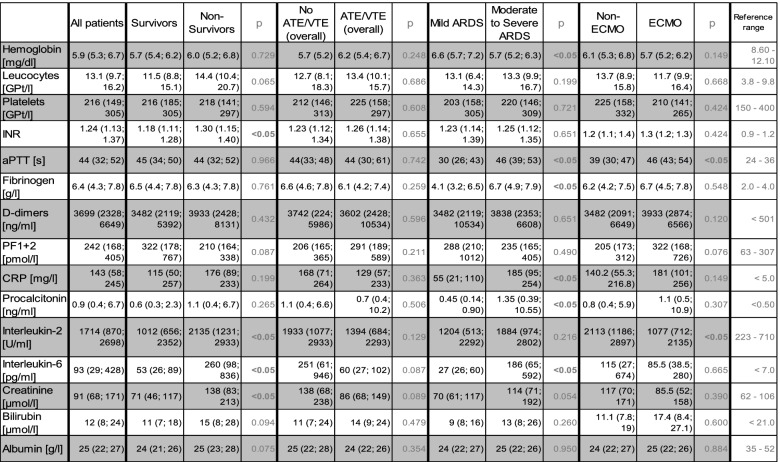
Laboratory results – Baseline and subgroup differentiation

*ATE/VTE* Arterial/Venous Thromboembolism, *ARDS* Acute Respiratory Distress Syndrome, *ECMO* Extracorporeal Membrane-Oxygenation, *INR* International Normalized Ratio, *aPTT* Activated Partial Thromboplastin Time, *PF1* + *2* Prothrombin Fragments 1 + 2, *CRP* C reactive protein

**Table 3.**
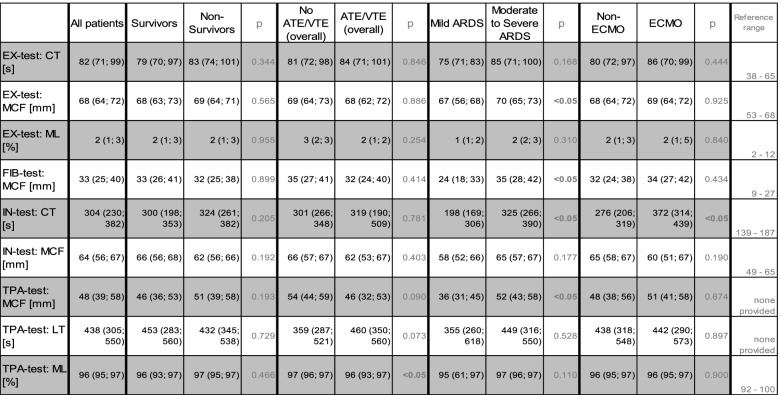
Viscoelastic testing results – Baseline and subgroup differentiation

*ATE/VTE* Arterial/Venous Thromboembolism, *ARDS* Acute Respiratory Ristress Syndrome, *ECMO* Extracorporeal Membrane-Oxygenation, *CT* Clotting Time, *MCF* Maximum Clot Firmness, *ML* Maximum Lysis, *LT* Lysis Time

### Survival and Complications during follow-up

Follow-up was completed in 100% of all patients for the duration in our ICU. Median follow-up duration was 25 days (range 0 – 67 days, 17; 33). Overall ICU-mortality was 55% (*n* = 30) in this cohort. Non-survivors were older (67 vs 61 years), showed higher SOFA-scores (12 vs 10 points), needed higher catecholamine doses (median 0.14 vs 0.07 μg/kg/min) and were noticed with significant lower median PaO_2_/F_I_O_2_ (45 mmHg vs 60 mmHg) in absence of ECMO therapy. No significant differences were found between survivors and deceased patients in terms of previous diseases, but permanent preexisting use of beta blocker drugs was higher in non-survivors. Patients who died during ICU stay showed significantly higher values for INR (median 1.30 vs 1.18), Creatinine (median 138 vs 71), IL2 (median 2135 vs 1023) and IL6 (median 260 vs 53). ClotPro® parameters and all other laboratory parameters did not significantly differ between groups. ECMO therapy was not associated with higher mortality (Table 4).

**Table 4:** Patients without ECMO compared to those with ECMO

	Non-ECMO	ECMO	p
Age [years]	69 (63; 71)	61.5 (56; 65)	** < 0.05**
Body Mass Index [kg/m^2^]	27.8 (25.1; 31.1)	31 (26.2; 37.5)	0.226
SOFA Score [points]	11 (10;14)	11 (10; 13)	0.910
Charlson Comorbidity Index	4 (3; 7)	2 (2; 3)	** < 0.05**
Dosis of UFH [mg/h]	1400 (800; 1800)	1600 (1200; 1900)	0.238
LMWH [mg/24 h]	120 (100; 180)	-	-
Horovitz Quotient at ICU admission ^*^ [mmHG]	146 (114; 219)	160 (137; 189)	0.487
Lowest Horovitz Quotient during ICU stay ^*^ [mmHG]	52 (45; 67.5)	53 (37.5; 67.5)	0.848
Prophylactic Anticoagulation	9 (27%)	3 (14%)	0.195
Intermediate Anticoagulation	11 (33%)	15 (68%)	** < 0.05**
Any coagulation-associated Complication	19 (58%)	12 (55%)	0.824
BARC_2	2 (6%)	2 (9%)	0.528
BARC_3c	1 (3%)	1 (5%)	0.999
VTE/ATE at VET	14 (42%)	8 (36%)	0.653
ATE	2 (6%)	0	0.356
DVT	8 (24%)	7 (32%)	0.376
CAT	0	2 (9%)	0.156
PE	11 (33%)	6 (27%)	0.634
In-hospital Death	20 (61%)	10 (45%)	0.269

*ECMO* Extracorporeal Membrane-Oxygenation, *SOFA* Sepsis-related organ failure assessment, *UFH* Unfractionated heparin, *LMWH* Low-molecular-weight heparin, *ICU* Intensive Care Unit, *BARC* Bleeding Academic Research Consortium, *VTE* Venous Thromboembolism, *ATE* Arterial Thromboembolism, *VET* Viscoelastic Testing, *DVT* Deep Vein Thrombosis, *CAT* Catheter-Associated Thrombosis, *PE* Pulmonary Embolismm, *ARDS* Acute Respiratory Distress Syndrome

At time of admission to our department, 40% of all patients (*n* = 22) presented with ATE/VTE. PE was diagnosed in 29% (*n* = 16), DVT in 20% (*n* = 11), CAT in 4% (*n* = 2) and ATE in 2% (*n* = 1) of all patients. Therapeutic anticoagulation was already administered in 64% (*n* = 14) of patients with ATE/VTE, while 23% (*n* = 5) received intermediate anticoagulation therapy and 14% (*n* = 3) only prophylactic anticoagulation. Patients with ATE/VTE before VET-measurement received significantly higher doses of unfractionated (median 1900 vs 1100) or low molecular heparin (median 140 vs 90, data not shown).

During stay on our ICU, only five patients developed additional ATE/VTE. In 4 of these patients DVT (80%) was diagnosed, one patient presented with PE (20%). One patient with newly diagnosed PE presented with additional ATE leading to immediate surgical thrombectomy of the radial artery. Thus, the total number of patients with ATE/VTE during the entire hospital stay was 27 (49%). The majority (22 patients, 40%) was diagnosed with ATE/VTE already at admission to our ICU. Only 9% of all patients (*n* = 5) were diagnosed with additional ATE/VTE during stay on our ICU.

18% (*n* = 10) of patients suffered from bleeding complications during follow-up. Bleeding complications were classified in 4 patients as BARC II, in 6 patients as BARC IIIb and 2 patients suffered from intracranial hemorrhage (BARC IIIc), none of patients suffered fatal bleeding complications according to BARC classification IV or V (Table 1). 60% of them showed concurrent ATE/VTE (*n* = 6).

In this study overall 56% of the patients (*n* = 31) had complications associated with disarranged coagulation or fibrinolysis – bleeding or ATE/VTE – with the following order: PE ± DVT (*n* = 17), lower extremity DVT (*n* = 7 patients), catheter associated thrombosis (*n* = 2) and ATE (*n* = 2). Interestingly, in only8 out of 17 (47%) patients with PE, a corresponding thrombosis could be found.

Patients with bleeding complications presented with a slightly prolonged aPTT (median 47 vs 43 s, *p* = 0.50), slightly shortened INR (median 1.20 vs 1.26, *p* = 0.63) and not significantly prolonged CT at IN-test (median 325 vs 300, *p* = 0.41) and CT at EX-test (94 vs 81, *p* = 0.40) compared to non-bleeding patients.

All patients with ATE/VTE during the entire ICU-stay showed notably prolonged, median LT 460 s vs 359 s (*p* = 0.073) and significantly lower ML (median 96% vs 97%, *p* < 0.05) in TPA assay as a sign of reduced fibrinolytic capacity. No significant changes could be found in D-dimers or PF 1 + 2 values, nor was the anticoagulation regime associated with any predictive value for ATE/VTE or bleeding (Tables 2 and 3).

### Coagulopathy in ECMO patients compared to non-ECMO patients

Patients under ECMO therapy were younger (median age 62 vs 69 years, *p* < 0.05) and had less complex comorbidities (Charlson Comorbidity score of 2 vs 4 points; *p* < 0.05). On admission to our ICU, patients within the ECMO subgroup had similar SOFA scores. ECMO patients received slightly higher doses of UFH, but most patients on ECMO therapy were on an intermediate anticoagulation regime, while therapeutic anticoagulation was more frequently provided to non-ECMO patients (18% ECMO vs. 39% non-ECMO, *p* = 0.095). DVT/PE tended to occur more frequently in non-ECMO patients (45% ECMO vs 52% non-ECMO, p = 0.660), while ECMO patients suffered non-significantly more from bleeding complications (32% ECMO vs 15% non-ECMO, *p* = 0.129). In our cohort, ECMO patients showed a comparable outcome to non-ECMO patients with in-hospital survival of 55% to 39% in non-ECMO COVID-19 ARDS patients (*p* = 0.269; Table 4). Differences of laboratory parameters at admission to our ICU were mostly related to increased anticoagulation therapy with prolonged aPTT (*p* < 0.05) and CT in IN-test (*p* < 0.05). PF 1 + 2 were non-significantly higher in ECMO patients with median 322 vs 206 pmol/l (*p* = 0.076 Tables 2 and 3).

### ClotPro® parameters and conventional laboratory assays for anticoagulation monitoring

In the assessment of the intrinsic coagulation pathway, values for aPTT correlated significantly with CT values of IN-test (r_s_ 0.561, *p* < *0.05*. Doses of administrated UFH were highly correlated with CT-values of IN-test (r_s_ 0.607, *p* < *0.05*) and values of aPTT (r_s_ 0.585, *p* < *0.05*). Assessment of the extrinsic coagulation pathway demonstrated that values for INR correlated significantly with CT values of EX-test (r_s_ 0.493, *p* < *0.05*). Values of functional plasma fibrinogen correlated strongly with MCF of FIB-test (r_s_ 0.855, *p* < *0.05*).

## Discussion

### Thromboembolic complications

We found a high incidence of ATE/VTE in invasively ventilated COVID-19 ARDS patients comparable to previous studies on COVID-19 patients in the ICU [6, 44–46] if screening was routinely performed at hospital admission [47]. Generally, these data are difficult to compare due inhomogeneous definitions of ATE/VTE. For instance, calf veins are routinely included in our institutional ultrasound protocols and catheter-related clots are also counted, whereas other institutions may not collect such data. Even the definition of pulmonary embolism varies between studies [50]. This should be taken into account when discussing incidences for ATE/VTE or effects of different anticoagulation strategies. Most of our patients with ATE/VTE presented with preexisting thromboembolic complications on the day of admission to our ICU. Although, increased dosage of anticoagulation could not prevent initial thromboembolisms, the incidence of new ATE/VTE after admission to our department was low (5 patients, 9%) compared with preexisting ATE/VTE at admission to our ICU (22 patients, 40%). Noteworthy, VTE rates in patients with severe influenza ARDS were considerably lower at 3% [51] compared to COVID-19 infected patients. Higher D-dimers and PF 1 + 2 values did not show any significant correlation with ATE/VTE in our cohort. Additional hypercoagulation in VET, defined as increased MCF in EX-test or FIB-test did not correlate with ATE/VTE, but with severity of ARDS at ICU admission. Patients with thromboembolic complications during their ICU stay showed a tendency of impaired fibrinolytic capacity in the ClotPro® TPA-assay. The clinical consequence of a slightly reduced maximum lysis and prolonged lysis time has to be interpreted with caution and put into context with the individual patient’s situation, since there is currently no evidence, that a reduced fibrinolytic capacity as a laboratory finding has a corresponding clinical correlate. Deceased patients showed higher values of IL-2, IL-6 and creatinine, were older and had higher organ dysfunction (SOFA) scores at ICU admission. Neither ClotPro® parameters nor D-dimers / PF 1 + 2 values had any predictive value considering ICU-mortality.

The prevalence of VTE is up to 50% in COVID-19 infected ICU-patients and considerable higher compared to non-COVID patients [6, 52–54], if screening is routinely performed at hospital admission [55]. Furthermore, VTE in COVID-19 patients seems to be associated with a higher mortality and is more likely among patients with severe ARDS (38%) [56].

### Severity of ARDS

Since not all COVID-19 infected patients with manifested PE showed coexisting DVT in autopsy studies, Ackermann et al. suggested an own entity of pulmonary thrombosis according to the vascular damage caused by COVID-19 without concomitant DVT [57]. The occurrence of an entity, described as singular pulmonary thrombosis might explain, why in nearly half of all patients in our study presenting with PE, DVT could not be detected by cCUS. These findings might underline the local intrapulmonary pro-thrombotic and hypofibrinolytic state in these patients, which cannot be fully explained by current concepts of systemic hypercoagulation or impaired fibrinolysis with distal thrombosis formation and subsequent embolic transfer to the lung arteries [58]. However, the pro-thrombotic and anti-fibrinolytic situation may explain the correlation between increased clot firmness in VET and progressed ARDS severity in our study. Particularly, in the ClotPro® TPA-assay – analyzing clot formation under the influence of tPA – increased clot firmness was strongly associated with ARDS severity. Possibly, this can be interpreted as a resistance to fibrinolysis, which might also be reflected in the slightly prolonged lysis time. To our knowledge, this is the first report correlating ARDS severity and VET parameters. Progress of the disease in COVID-19 patients leads to severe micro thrombotic complications, which cannot be detected easily by current clinical practice or any of the conventional laboratory tests. This highlights the importance for blood coagulation analyses by VET which might be useful in identifying a presumed hypercoagulable microthrombotic condition in these patients. Al-Samkari showed in a recent study, that PF 1 + 2 values were more specific in predicting ATE/VTE in COVID-19 patients than D-dimer level [59] and further studies strongly recommend PF 1 + 2 for the diagnosis of thrombosis [60, 61]. In contrast, PF 1 + 2, as markers of direct thrombin activation, did not correlate significantly with overall ATE/VTE in our study. Coagulation parameters at admission to our ICU did not differ extensively between ECMO and Non-ECMO patients, except for PF 1 + 2. Hundalani et al. reported in a series of 29 pediatric patients under ECMO therapy that markers of hypercoagulation, e.g. PF 1 + 2 as well as thrombin-antithrombin-complex, plasmin-antiplasmin complex and d-dimers, increased from day 1 to day 5 [62]. Increased values of PF 1 + 2 might be caused by the activation of the coagulation system due to the ECMO circuit and should therefore not generally be taken into account as predictive value for ATE/VTE.

One advantage of using VET over conventional laboratory testing in critically ill patients might be the early recognition of hypercoagulability. This was demonstrated n postoperative patients or prostate cancer patients [63]. In our study we could not show a strong correlation to forecast ATE/VTE on the base of VET results or laboratory analysis. This might be explained by the advanced stage of COVID-disease in patients already presenting with hypercoagulation and subsequent strong anticoagulation therapy before enrollment in the study.

### Predictive ability of VET compared to conventional laboratory testing for short-term outcome

**Fig. 3:**
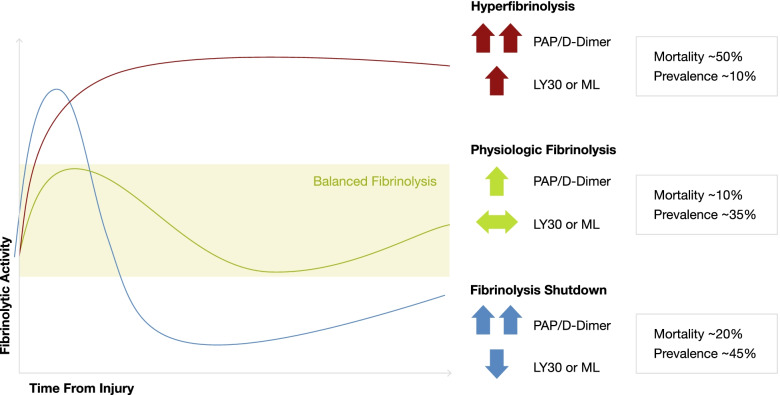
Relation between Fibrinolytic Activity and D-dimers / VET values. Adapted from Moore et al. [35]

High D-dimer levels and their rapid rise could be demonstrated as valid predictors of mortality in COVID-19 [64–66] and septic patients [67]. D-dimers concentration > 0.5 mg/l (> 500 ng/ml) at hospital admission was significantly associated with increased 30-day mortality in another study [68]. In our study there was no significant association between D-dimer values and ATE/VTE, mortality or ARDS severity. Of note, the origin of D-dimers is complex and elevated D-dimers do not necessarily indicate enhanced fibrinolysis because large extravascular deposits of fibrin may also increase D-dimer levels [69]. Especially in COVID-19 ARDS the lung might be the most important source of D-dimers [28], for instance due to fibrin deposits within pulmonary alveoli. Furthermore, elevation of D-dimers, PF 1 + 2, FDP and PAP level as byproducts of clot lysis can be caused by increased activity of the plasmin system, increased clot charge from severe tissue injury, intravascular thrombosis, disseminated intravascular coagulation or even decreased product clearance. Therefore, high D-dimer values do not necessarily reflect systemic fibrinolytic activity [35]. Moreover, elevated levels of plasmatic D-dimers are not only dependent on fibrinolysis but also on coagulation [70]. Moore et al. assumed different phenotypes of fibrinolysis responders after traumatic injury, in terms of hyperfibrinolysis, physiologic fibrinolysis or fibrinolysis shutdown [71]. D-dimers or PAP values could not contribute to distinguish between phenotypes, whereas VET might provide new insights (Figure 3) [71]. Evidence suggests that even increased markers of coagulation and fibrinolytic activation can be found in septic patients without correlation with impaired fibrinolytic activity measured by VET or other global fibrinolysis tests [63]. Equally, patients under ECMO showed onlya poor correlation of D-dimer values and hypercoagulability measured by VET [72]. In the study by Bachler et al. D-dimer levels were useful in predicting impaired fibrinolysis in contrast to VET [40].

**Fig. 4:**
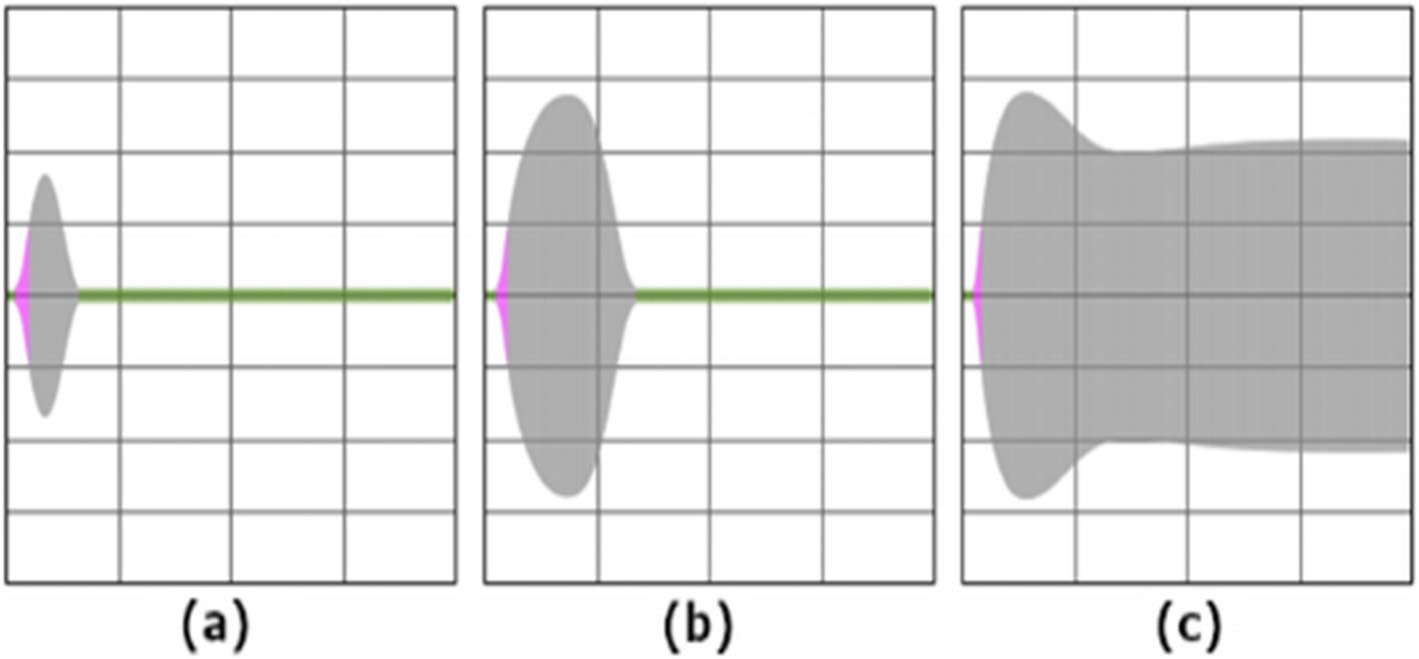
Typical display of results of TPA assay in **a** healthy patients, [(b) + (c)] with severe cARDS. Sign of hypofibrinolysis **b** up to fibrinolytic shutdown **c** has been observed frequently. X-axis is time and amplitude is mechanical delay of rotational speed caused by clotting of sample

Limited evidence suggests that the use of VET, in particular in combination with D-dimer values and CRP, may help predicting ATE/VTE [29, 41, 73, 74]. Bachler et al. [40] proposed in their study comparing critical ill patients with COVID-19 to healthy controls, that impaired fibrinolysis should be assumed if lysis time is ≥ 393 s. Our results suggests, that prolonged lysis time in combination with reduced maximum lysis should be considered as a predictor for patients at risk for ATE/VTE. Regarding the limited number of cases with ATE/VTE during their stay at our ICU, this study does not have enough statistical power to provide reliable cut-off values for LT in ROC analysis. Notably, the American College of Surgeons and the American Society of Hematology included VET in their recommendations for the management of COVID-19 related coagulopathy [75]. In January 2021, the United States Food and Drug Administration (FDA) also recommended VET for monitoring coagulation of COVID-19 patients [76]. Clinical observation suggests, that in some patients progressive impaired fibrinolytic capacity might be associated with higher mortality and should be critically observed (Figure 4). However, in our study no statistically significant evidence could be found between mortality and results of VET. Therefore, our hypothesis may need to be re-evaluated in patients suffering from earlier stages of COVID-19. Therefore, we would recommend to use specific assays (tPA-test) to analyze fibrinolytic capacity, when performing VET in patients at risk for ATE/VTE.

### Bleeding events

Approximately 8 – 18% of COVID-19 patients are reported to suffer from bleeding events, mainly in the gastrointestinal tract [77, 78]. The balance between bleeding and ATE/VTE remains a challenge in the treatment of ICU patients, particularly in the setting of invasive procedures Recent publications demonstrated safe performance of invasive procedures using rotational VET. While aPTT was prolonged, VET showed increased or normal clot formation [79, 80]. The aPTT does not reflect the balance of pro and anticoagulant clotting factors, but CT in VET assays might do. This could be due to the fact that closer to physiological conditions are found in wholeblood testing compared to testing for isolated biochemical reactions found in the complex coagulative system. However, we reported a high incidence of bleeding complications (18%) in our cohort. None of VET or laboratory parameters could significantly predict these bleedings complications. The non-significantly prolonged CT in EX- and IN-test in bleeding patients compared to normal aPTT and INR requires further investigations.

In our study bleeding complications were non-significantly higher in patients under ECMO therapy. Bleeding complications, in particular intracranial hemorrhage is frequent in patients with need of ECMO therapy [81]. A recent multicenter study analyzing 210 patients under ECMO therapy with/without COVID-19 suggested, that intracranial hemorrhages (ICH) are 3.5-fold increased in COVID-19 patients under ECMO therapy [82]. However, in our study only two patients (1 under ECMO therapy, 1 without ECMO therapy) suffered from ICH. The high incidence of bleeding complications during ECMO therapy as well as in non-ECMO patients in the current study might rather be caused by increased anticoagulation as standard approach in all ICU patients than by ECMO-related coagulopathy. As mentioned above according to the institutional SOP, patients admitted to ICU received subtherapeutic unfractionated heparin (target aPTT of 40-50 s) or intermediate doses of low molecular weight heparin (100 aXa units/kg/d). Although this is not completely in line with the current German Guideline, the authors decided in an interdisciplinary approach to proceed in the mentioned way.

### VET in COVID-19 associated coagulopathy

The results of our study investigating coagulopathy using ClotPro® in COVID-19 patients, do not support the results of other studies. Bachler et al. analyzed 20 COVID-19 patients with moderate disease severity (mean SOFA score 6.5 points) and compared their results to healthy individuals [40]. This group also evaluated hypercoagulability and fibrinolysis using a newly developed TPA-test. Infanger et al. investigated 27 more severe COVID-19 patients (mean SOFA score 11 points), often requiring mechanical ventilation but without ECMO-support [48]. In this study ClotPro® parameters were compared to ROTEM® Delta parameters and conventional laboratory assays, TPA-test was not performed. Compared to our study, hypercoagulability at baseline was nearly similar (median MCF in EX-test of 68 mm in our study to median of 68.5 mm (Bachler et al. [40]) and mean of 70 mm (Infanger et al. [48]). However, considerable discrepancies were found with regard to the effect of anticoagulants or deficiency of coagulation factors. For instance, median CT in EX-test was 82 s in our study cohort but only 51.5 s (Bachler et al. [40]) or 77 s (Infanger et al. [48]), respectively. In addition, median CT in IN-test was 304 s at baseline in our study compared to 188 s (Bachler et al. [40]) and 165 s (Infanger et al. [48]), respectively. In those previous studies, thromboprophylaxis was mainly based on LWMH, which may explain the lack of CT prolongation in IN-test but not the differences in EX-test. Whether COVID-19 disease severity might increase CT in EX-test, especially if patients are additionally exposed to extracorporeal circulation remains speculative.

### Study limitations

It is important to emphasize that the interpretation of VET results in Covid-19 patients is still under discussion. Beside the fact that the results of different VET devices are not always interchangeable (due to different technologies). Furthermore, there are no clear cut-off values for the diagnosis of neither clinically relevant hypercoagulability nor hypofibrinolysis [83]. Another limitation of our study could be that all blood samples were drawn from the arterial line instead of the venous line of our ICU patients. (routine procedure in many ICUs). We need to point out that, at least in theory, VET results may vary between venous and arterial samples, due to the differences in cell activation from shear stress. Any such errors would be systematic and not interfering with the internal validity of our findings or the group comparisons, but may affect the validity of our findings if compared to studies using venous samples.

As with all retrospective studies, limitations and biases include selection bias, the risk of underreporting and the inability to demonstrate causal relationships. At best, our results can be regarded as hypothesis-generating. We observed different variations in patient characteristics and quantities that are likely to have an effect on prognosis. The main bias in this study is the inhomogeneous disease stage and pre-treatment phases, caused by a high number of patients admitted from other hospitals or ICUs.. To improve homogeneity of the study group, we enrolled only mechanically ventilated patients. Hence, we report on a subset of critically diseased patients with a median SOFA-score of 11 and 40% requiring ECMO support. Across this population, we recorded pronounced elevations of D-dimer (median 3699, range 887 – 20,000) and median PF 1 + 2 values (242, range 65 – 3590) which might limit the explanatory power. Additionally, not all samples could be accurately titrated since some patients presented with D-dimer and F1 + 2 values exceeding the upper range of detection. The aim of this study was to evaluate the benefit of different laboratory parameters and VET in critical ill patients at high risk for mortality and ATE/VTE. Hence, we did not compare our results to healthy controls or historic control group. The objective of this study was to identify parameters with predictive ability for short term outcome in a cohort of critical ill patients. Finally, the number of thromboembolic events during the stay in our ICU was lower than expected, which limits the power of statistical analysis. Notably, 40% of our patients presented with ATE/VTE on the day of the first measurement on admission with an increased anticoagulation therapy already established.

In addition, there are some limitations arising from technical restrictions, e.g. effect of platelet dysfunction and abnormality of von Willebrand factor (vWF) level which were not analyzed. Furthermore, effects of blood flow and endothelial damage are neglected [63].

## Conclusion

Regarding the complexity of the coagulopathy in cARDS patients, conventional laboratory analyses might not be feasible to identify patients at risk for ATE/VTE. Microthrombi of the pulmonary arteries besides PE may aggravate ARDS and are difficult to detect. In our study we could show that patients with increased clot firmness were more likely to present severe ARDS, whereas D-dimers and PF 1 + 2 did not differ significantly. Furthermore, patients with reduced fibrinolytic capacity should be considered at risk for ATE/VTE. Mortality was not predictable by VET, D-dimers or PF 1 + 2 values, while inflammatory cytokines such as IL-2 and IL-6 were significantly correlated with increased mortality.

POC coagulation testing provided by VET might help to detect a hypercoagulation state and impaired fibrinolysis in critically ill ARDS patients. The clinical impact of VET to optimize diagnostics of coagulopathy in various disorders requires further investigation.

## Data Availability

The datasets are not publicly available due to data sharing protocols but are available from the corresponding author on reasonable request.

## Abbreviations

A5: Thickness of clot amplitude 5 min after clotting time
A10: Thickness of clot amplitude 10 min after clotting time
ACE-2: Angiotensin-converting enzyme 2
aPTT: Activated partial thromboplastin time
ARDS: Acute respiratory distress syndrome
ATE/VTE: Thromboembolic complications
AT-III: Antithrombin-III
aXa: Anti-Xa activity
cARDS: COVID-19 associated acute respiratory distress syndrome
CAT: Catheter associated thrombosis
CRP: C-reactive protein
CFT: Clotting formation time
CT: Clotting time
CTPA: Computed tomography pulmonary angiography
DTI: Direct thrombin inhibitors
DVT: Deep vein thrombosis
ECMO: Extracorporeal membrane oxygenation
ICU: Intensive Care Unit
IL: Interleukin
INR: International normalized ratio
LT: Lysis Time
MCF: Maximum Clot Firmness
ML: Maximum Lysis
PAI: Plasminogen activator inhibitor
PAP: Plasmin-antiplasmin complex
PE: Pulmonary Embolism
PF 1 + 2: Prothrombin fragment 1 + 2
POC: Point-of-care
PT: Prothrombin time
RAAS: Renin–angiotensin–aldosterone system
RVV-V: Russell viper venom factor V
rTF: Recombinant tissue factor
TAT: Thrombin-Antithrombin-Complex
TF: Tissue factor
tPA: Tissue plasminogen activator
uPA: Urokinase plasminogen activator
VET: Viscoelastic testing
VTE: Venous thromboembolism
vWF: Von Willebrand factor
WHO: World Health Organization
